# Effectiveness of 36 hospital learning centers in Thailand: continuation of child patient education, parent attitudes toward child’s illness and service satisfaction

**DOI:** 10.12688/f1000research.26599.1

**Published:** 2020-12-11

**Authors:** Adidsuda Fuengfoo, Kim Sakulnum, Sumitra Owjinda

**Affiliations:** 1 Division of Developmental and Behavioral Pediatrics, Department of Pediatrics, Queen Sirikit National Institute of Child Health, Bangkok, Thailand; 2 College of Medicine, Rangsit University, Bangkok, Thailand; 3 Division of Child Early Stimulation, Department of Nursing, Queen Sirikit National Institute of Child Health, Bangkok, Thailand

**Keywords:** child illness, education service, school engagement, satisfaction, hospital learning center

## Abstract

**Background:** This study aimed to determine the effectiveness of 36 hospital learning centers for the continued education of sick children using electronic distance learning television (eDLTV), parents’ attitudes toward their child’s illnesses and education, and service satisfaction of the centers.

**Methods:** The sample included 4,430 children aged 4-18 years old with common illnesses, chronic illnesses and developmental disorders, as well as 4,430 parents who had taken care of the child for at least 6 months. The methods included attitude surveys, which were analyzed using chi-square tests and t-tests.

**Results:** The factors associated with education continuation of the children were illness types (parents were less worried about children with common illness and more concerned about education of children with chronic diseases and children with disabilities), distance from home to school, transportation type, parents’ education level, marital status, and family income. About 99.8% of patients with common illnesses continued their education, followed by 99.3% of disabled children, and 95.9% of chronic patients. Satisfaction score towards the services at the learning centers were high (mean scores: 4.28 and 4.43 respectively, out of 5 = strongly satisfied).

**Conclusion:** After completing an education program through eDLTV at a center, a total of 97.7% of children continued their education and were highly satisfied with the service at the center. Parents had positive attitudes towards their child’s illnesses and education.

## Introduction

As a crucial foundation for development, education should be equally accessible to all children, regardless of differences in social or physical status. Today medical advancements have increased the survival rate of children with chronic diseases to 90%, reducing mortality rates and complications ^1^, and increasing life expectancy by as much as 20 years longer ^2^. The prevalence of children with chronic diseases differs depending on definition. A survey in the United States indicated that approximately 20% of children had chronic illnesses ^3^. In Thailand, the number is still unclear because chronic illness is defined as a long-term illness involving treatments that might affect the lifestyles of children and families ^4– 6^. Such changes can include absence from school, which could affect learning and social activities ^7, 8^. 

The UN Convention of the Rights of the Child states that every child has the right to be protected and receive equal education ^9^. As a member of the UN, Thailand implemented a law stipulating that “education is not only limited to the classroom”. Therefore, medical institutes have merged with special education centers to establish “learning centers in the hospital” so that pediatric patients can continue to have equal access to education. Making use of information technology to support education, the project is called “The Information Technology Project under the Initiative of Her Royal Highness Princess Maha Chakri Sirindhorn”. Similar learning centers in other countries involve the coordination of multidisciplinary teams to enhance social and learning activities or sick children.

The Queen Sirikit National Institute of Child Health (QSNICH) Learning Center in Thailand has been operating for 20 years. In the past ^10^, education for sick children supported academic knowledge and daily living skills which made them feel included in society, and reflected educational and social perceptions of the related life adjustments and psychological conditions. QSNICH has previously conducted research ^11^ on education for sick children, aiming to determine the effectiveness of learning centers in 36 provinces in Thailand. With official ministry-level collaboration among various parties to obtain operational results regarding continued learning for sick children and the associated factors ^12^, QSNICH has provided a model for other hospitals across Thailand to ensure that all sick children have equal access to education.

### Objectives

This study has been extended from previous research ^13^ on the effectiveness of the learning center at QSNICH, which investigated the associated factors affecting further education of children with special needs. The aims of this study were to determine the effectiveness of 36 other network learning centers across Thailand for the continued education of pediatric patients, as well as the factors associated with the patients’ further education. It also evaluated parents’ attitudes toward their children’s illness and education, as well as service satisfaction of the center.

## Methods

### Study design

A cross-sectional study was conducted to collect information from the target population at 36 learning centers in the hospitals across Thailand under the supervision of Thai Ministry of Public Health. The study used a survey to collect information ( *Extended data* ^14^). The study was conducted from January 1, 2018, to December 31, 2018.

### Participants

The target population included 4,430 child patients aged 4-18 years as well as 4,430 parents who had taken care of the child for at least six months, meaning that the total study population included 8,860 individuals. The sample size calculation was based on Wayne ^15^. The total number of pediatric patients from 36 learning centers at the hospitals was 30,000 (n=30,000), and the incidence of children who went back to school was 80% (p=0.8))

Systematic random sampling was used to select parents and children from the lists of patients at the 36 learning centers. If any individual refused to participate, the next name would be selected.

The survey was conducted by the researchers after the parents gave written informed consent for their child to participate. For children aged 4–7 years, parents were asked to participate in the survey on behalf of the child. The session was audio recorded for data analysis.

### Eligibility criteria

Inclusion criteria: 1) Pediatric patients age between 4–18 years who are attending learning centers at the hospital; 2) Pediatric patients who have attended learning centers more than 2 times; 3) pediatric patients with chronic illnesses who are admitted at the hospital or needed consecutive follow-up.

Exclusion criteria: pediatric patients with serious illness and life-threatening conditions.

### Data collection

The survey was divided into four parts: (1) demographic information of the children and parents (gender, age, cause of admission and length of stay ( Table 1)), (2) information about the patient-parent relationship, and (3) satisfaction with service at the learning centers (measured using Likert scales of 1=unsatisfied, 2=poor, 3=fair, 4=satisfied, 5=very satisfied).

**Table 1.** Demographic information about the child patients.

	Common illnesses	Chronic illnesses	Developmental disorders	All
Total, n	1764	2384	282	4430
Gender, n (%)				
*Male*	952 (53.97)	1244 (52.18)	196 (69.50)	2392 (54.00)
*Female*	812 (46.03)	1140 (47.82)	86 (30.50)	2038 (46.00)
*Missing data*	*0 (0.00)*	*0 (0.00)*	*0 (0.00)*	*0 (0.00)*
Age (years)				
Mean (SD)	8.91 (3.30)	9.81 (3.41)	9.07 (3.01)	9.40 (3.37)
Median (IQR)	8.69 (6.17 - 11.19)	9.65 (7.10 - 12.44)	8.85 (6.90 - 11.11)	9.21 (6.76 - 11.86)
Min, max	2.68, 19.91	3.11, 20.89	3.00, 20.21	2.68, 20.89
*Missing data, n (%)*	*3 (0.17)*	*0 (0.00)*	*2 (0.71)*	*5 (0.11)*
*CAUSES OF ADMISSION*				
Common diseases, n (%)				
*Respiratory system e.g. pneumonia bbronchitis*	284 (16.10)			284 (6.41)
*Neurological disorder e.g. seizure*	53 (3.00)			53 (1.20)
*Gastrointestinal disorders (e.g. enteritis,* *stomachache, vomiting)*	487 (27.61)			487 (10.99)
*Accident*	213 (12.07)			213 (4.81)
*Urinary system (e.g. Urinary Tract Infection, acute* *pyelonephritis)*	128 (7.26)			128 (2.89)
*Infection (e.g. dengue fever, Influenza, encephalitis)*	328 (18.59)			328 (7.40)
*Other types of illness, e.g. fever, operation,* *appendicitis*	294 (16.67)			294 (6.64)
Chronic diseases				
*Heart disease*		119 (4.99)		119 (2.69)
*Cancer*		526 (22.06)		526 (11.87)
*Kidney disease*		153 (6.42)		153 (3.45)
*Blood disorders*		1055 (44.25)		1055 (23.81)
*Chronic lung diseases/ pneumonitis /asthma*		265 (11.12)		265 (5.98)
*Ulcerative colitis/gastritis*		29 (1.22)		29 (0.65)
*Neurological disorder/ epilepsy / tumors*		106 (4.45)		106 (2.39)
*Diabetes*		61 (2.56)		61 (1.38)
*High blood pressure*		7 (0.29)		7 (0.16)
*Other, e.g. lupus, allergies, bone diseases*		90 (3.78)		90 (2.03)
Developmental disorders, n (%)				
*Learning disability*			56 (19.86)	56 (1.26)
*Attention deficit hyperactivity disorder*			92 (32.62)	92 (2.08)
*Autism*			33 (11.70)	33 (0.74)
*Intellectual disability*			90 (31.91)	90 (2.03)
*Other types of developmental disorder, e.g. cerebral* *palsy, literacy, motion disorders*			30 (10.64)	30 (0.68)
Length of stay, n (%)				
*1–5 days*	1222 (69.27)	1225 (51.38)	235 (83.33)	2682 (60.54)
*6–10 days*	448 (25.40)	514 (21.56)	33 (11.70)	995 (22.46)
*11–15 days*	51 (2.89)	223 (9.35)	3 (1.06)	277 (6.25)
*> 15 days*	43 (2.44)	422 (17.70)	11 (3.90)	476 (10.74)
*Missing data*	*0 (0.00)*	*0 (0.00)*	*0 (0.00)*	*0(0.00)*

### Statistical analysis

SAS 9.4 software was used for data management. Descriptive statistics, including percentages, means, and standard deviations, were used to analyze the population data and SAS 9.4 software was used to calculate frequency, percentage, mean, and standard deviation to measure the attitudes and satisfaction of the parents and children. Satisfaction data were divided into five levels, from strongly agree to strongly disagree, based on the statistical test results. Chi-square tests and t-tests were used to assess the variables associated with education continuance at the learning center.

### Ethics statement

This study and the selection of the participating hospitals were approved by the Office for Ethics in Human Research, Ministry of Public Health, Thailand in 2018 (ref. REC.024/2561), and was conducted in accordance with the Declaration of Helsinki.

## Results

### Caregiver information

The patient population was classified into three groups: those with common illnesses, chronic illnesses, and developmental disorders (n=4,430). There were a total of 4,430 caregivers: 82.94% were caregivers for patients with common illnesses, 82.68% chronic illnesses, and 72.34% developmental disorders. Biological parents or relatives comprised 17.07% of the common illnesses group, 17.28% chronic illnesses, and 26.95% development disorders; foster parents or teachers accounted for 0.06%, 0.04%, and 0.71%, respectively. Most caregivers were women (75.74%, 80.83%, and 82.62%, respectively), of Thai nationality (99.26%, 99.33%, and 99.65%, respectively), and Thai ethnicity (97%, 98.83%, and 99.65%, respectively). Other ethnicities included Akha, Lahu, and Burmese. The main religion was Buddhism (92.01%, 94.51%, and 97.87%, respectively), followed by Islam and Christianity. Most caregivers were aged 31–40 years (43.71%, 39.89%, and 34.40%, respectively), followed by those aged 41–50 years (23%, 39%, and 40%, respectively) and 21–30 (17.29%, 13.7%, and 18.86%, respectively). Most parents had completed vocational level, or high -school level education, followed by those with a primary or secondary educational level. Most parents were employed in labor (41.5%), agriculture (23.09%), and sales (11.22%). Most parents were married, separated, or divorced and had monthly household incomes of 10,0000–30,000 baht, followed by 5,000-10,000 baht; only a small number had incomes of >30,000 baht or <5,000 baht. Most families owned a house, while some rented a house or room. Only a small number lived with a relative. Most repondents lived in the northeastern part of Thailand.

### Parent-child relationship

Of the 4,430 patients, 1,764 patients had common illnesses, 2,384 chronic illnesses, and 282 developmental disorders. In general, the caregivers of all three types of patients rated the following issues similarly. Regarding illness severity, 29.9% rated it at the minimum level, 33.6% moderate, and 20% somewhat high. Regarding when the child became ill, 43.8% of caregivers always informed teachers while 24.02% often did so. Approximately 35.7% were somewhat highly confident that their child could get along with their peers, while 24.0% were somewhat confident and 24% were highly confident. Likewise, 37.8% of caregivers put somewhat high effort to into helping their child, while 25.0% made a high-level effort. Approximately, 37.6% frequently explained the assignments to their child, and 27.5% always did so. About 37.6% had high confidence that the school provided appropriate study plans for their child, 25.5% were moderately confident, and 26.1% were extremely confident. About 34.6% of patients discussed what happened in school with their child, and 23.3% did so all the time. Regarding caregivers’ confidence in encouraging their child to control their emotions, 37.6% were somewhat highly confident, 23.5% moderately confident, and 19.6% extremely confident. About 84% of caregivers sent their child to school every day.

During hospitalization, 25.1% of the patients were in kindergarten, 58.7% in primary school, and 13.8% in secondary school. After discharge, 22.3% in kindergarten, 57.5% in primary school, and 15.6% in secondary school. About 20.9% of the patients lived <1 km from school, 53.4% lived 1–5 km away, and 25.3% lived the same distance as before joining the learning center. About 57.0% traveled to school by bicycle/motorcycle, 17.3% by private car, and 20.0% by public transportation; the means of transportation remained almost the same before and after being treatment at the center.

### Children with common illnesses

As shown in Table 2, 28.5% of common illness patients were in kindergarten, 57.1% in primary school, and 13.2% in secondary school. After discharge, 28.0% were in kindergarten., 57.3% in primary school, and 13.3% in secondary school. Approximately, 15.7% lived 1 km or less from school, 56.8% 1– 5 km, and 27.5% the same distance as before entering the center. About 56.4% traveled to school by bicycle/motorcycle, 16.9% by personal vehicle, and 21.5% by public transportation. Transportation type remained largely the same before and after the treatment at the center.

**Table 2.** Educational background before and after entering learning centers, categorized by illness type.

	Before entering the center	After entering the center
	n (%)	N (%)
Children with common illness		
Total	1764	1764
Level of education		
*Kindergarten*	502 (28.46)	494 (28.00)
*Primary school*	1007 (57.09)	1010 (57.26)
*High school*	232 (13.15)	235 (13.32)
*Vocational education*	3 (0.17)	3 (0.17)
*Nonformal education*	17 (0.96)	18 (1.02)
*Other types of education, e.g. YMCA Child Development Center*	3 (0.17)	0 (0.00)
*Missing data*	*0 (0.00)*	4 (0.23) *
Distance from home to school, km		
*<1*	277(15.70)	275(15.59)
*1–5*	1001(56.75)	1001(56.75)
*>5*	485(27.49)	484(27.44)
*Missing data*	*1(0.06)*	*4(0.23)*
Transportation to school		
*Walk*	78(4.42)	78(4.42)
*Bicycle/ motorcycle*	995(56.41)	990(56.12)
*Personnel vehicle*	298(16.89)	297(16.84)
*Car hire/ school bus*	380(21.54)	382(21.66)
*Other types of transportation, e.g. public transport or attending a boarding school*	12(0.68)	13(0.74)
*Missing data*	*1 (0.06)*	*4 (0.23)*
Children with chronic illness		
Total	2384	2384
Level of education		
*Kindergarten*	556 (23.32)	440 (18.46)
*Primary school*	1420 (59.56)	1361 (57.09)
*High school*	354 (14.85)	429 (17.99)
*Vocational education*	8 (0.34)	9 (0.38)
*Nonformal education*	16 (0.67)	40 (1.68)
*Other, e.g. stopping school temporarily, attending nursery or a child development center*	30 (1.26)	7 (0.29)
*Missing data*	*0 (0.00)*	*98 (4.11)* ^*a*^
Distance from home to school, km		
*<1*	579 (24.29)	548 (22.99)
*1–5*	1224 (51.34)	1161 (48.70)
*>5*	565 (23.70)	575 (24.12)
*Missing data*	*16 (0.67)*	*100 (4.19)*
Transportation to school		
*Walk*	105 (4.40)	82 (3.44)
*Bicycle/ motorcycle*	1389 (58.26)	1345 (56.42)
*Personnel vehicle*	417 (17.49)	411 (17.24)
*Car hire/ school bus*	439 (18.41)	428 (17.95)
*Other, e.g. public bus, parent pick-up/drop-off*	18 (0.76)	19 (0.80)
*Missing data*	*16 (0.67)*	*99 (4.15)*
Developmental disorders		
Total	282	282
Level of education		
*Kindergarten*	55 (19.50)	52 (18.44)
*Primary school*	173 (61.35)	176 (62.41)
*High school*	27 (9.57)	27 (9.57)
*Vocational education*	0 (0.00)	0 (0.00)
*Nonformal education*	2 (0.71)	4 (1.42)
*Other, e.g. special education center, nursery or no education*	25 (8.87)	21 (7.45)
*Missing data*	*0 (0.00)*	*2 (0.71) ^*a*^*
Distance from home to school, km		
*<1*	68 (24.11)	66 (23.40)
*1–5*	142 (50.35)	141 (50.00)
*>5*	70 (24.82)	73 (25.89)
*Missing data*	2 (0.71)	2 (0.71)
Transportation to school		
*Walk*	16 (5.67)	15 (5.32)
*Bicycle/ motorcycle*	142 (50.35)	142 (50.35)
*Personnel vehicle*	52 (18.44)	53 (18.79)
*Car hire/ school bus*	65 (23.05)	65 (23.05)
*Other, e.g. attending a boarding school*	5 (1.77)	5 (1.77)
*Missing data*	*2 (0.71)*	*2 (0.71)*

^a^Did not study = 34 cases, Study drop = 70 cases

### Children with chronic illnesses

Before entering the center, 23.3% were in kindergarten, 59.6% in primary school, and 14.9% in secondary school. After discharge, 18.5% were in kindergarten., 57.1% in primary school, and 18.0% in secondary school. About 24.3% of patients lived less than 1 km from school, 51.3% 1–5 km, and 23.7% the same distance as before entering the center. About 58.3% traveled to school by bicycle/motorcycle, 17.5% by personal car vehicle, and 18.4% by public transportation. Means of transportation were mostly the same before and after treatment.

### Children with developmental disorders

Before entering the center, 19.5 % of patients were in kindergarten, 61.4% in primary school, and 9.6% in secondary school. After discharge, 18.4% were in kindergarten, 62.4% in primary school, and 9.6% in secondary school. About 24.1% of the patients lived less than 1 km from school, 50.4% 1–5 km. away, and 24.8 the same distance as before. About 50.4% traveled to school by bicycle/motorcycle, 18.4% by private vehicle, and 23.1% by public transportation. The means of transportation were slightly different before and after entering the center.

As shown in Table 3, satisfaction surveys were distributed to two groups of patients: those who continued their education and those who did not. Scores were classified into five ranges (1.00-1.80 = strongly disagree, 1.81-2.60 = disagree, 2.61-3.40 = uncertain, 3.41-4.20 = agree, and 4.21-5.00 = strongly agree). The average satisfaction score of the two groups was 4.21-5.00. Patients who continued their education thought that the lessons in the classroom were interesting; they scored this item higher than the other group did. Entertainment had been used to provide amusing and relaxed learning for these children. The patients who did not continue their education were those in final stage of life.

**Table 3.** Mean scores and standard deviations for service satisfaction at the center from the patient satisfaction survey.

Satisfaction	Continued education		Did not continue education	
	Mean	SD	Mean	SD
I am learning interesting lessons at the center	4.31	0.52	4.48	0.64
I enjoy learning new things at the center	4.32	0.52	4.45	0.54
I look forward to receiving services at the center	4.27	0.55	4.38	0.61
The atmosphere and facilities make me want to learn	4.27	0.55	4.40	0.57
There are various up-to-date learning materials available for me.	4.26	0.59	4.38	0.67
I am satisfied with the center	4.26	0.59	4.50	0.65
Total score	4.28	0.55	4.43	0.61

Table 4 shows the factors associated with education continuation. About 99.8%, 95.9%, and 99.3% of common, chronic, and development disorder patients continued their education, respectively. Significantly fewer chronic patients continued their education compared to common illness patients (OR=0.05; 95% CI: 0.02-0.14). About 97.8%, 97.5%, and 97.9% of kindergarten, primary school, secondary school students continued their education, respectively. There were no statistically significant differences between the three groups. Regarding distance from home to school, 97.1% <1 km away, 97.7% 1–5 km, and 98.7% >5 km; there were no statistically significant differences among the three groups. Regarding transportation to school, about 95.0% walked, 97.7% traveled by bicycle/motorcycle, 97.5% used a personal car, and 99.0% used public transportation. For those who continued their education, there were significant differences between patients who traveled by bicycle/motorcycle (OR=2.292; 95% CI: 1.152-4.560) and public transportation (OR=5.144; 95% CI: 2.062-12.833).

**Table 4.** Factors related to education continuation at the learning centers by the patients.

	Continue education	Discontinue education	Odds ratio (95% CI)	p-value
	n (%)	n (%)		
Child’s illness				
*Common illness*	1760 (99.77)	4 (0.23)	1	<0.0001*
*Chronic illness*	2286 (95.89)	98 (4.11)	0.05 (0.0 - 0.14)	
*Development disorders*	280 (99.29)	2 (0.71)	0.3 (0.06 - 1.75)	
Educational level (before entering learning center)				
*Kindergarten*	1089 (97.84)	24 (2.16)	1	0.7248
*Primary school*	2535 (97.50)	65 (2.50)	0.860 (0.535- 1.380)	
*Secondary school*	702 (97.91)	15 (2.09)	1.031 (0.537- 1.980)	
Length of hospital stay (days)				
*<5*	35 (100)	0 (0)	1	0.0392*
*5–10*	106 (97.72)	5 (2.28)	1.289 (0.807- 2.059)	
*>10*	44 (63.76)	15 (21.73)	2.217 (1.172- 4.194)	
Distance to school (km)				
*<1*	897 (97.08)	27 (2.92)	1	0.0492*
*1–5*	2313 (97.72)	54 (2.28)	1.289 (0.807 - 2.059)	
*>5*	1105 (98.66)	15 (1.34)	2.217 (1.172 - 4.194)	
Transportation to school				
*Walk*	189 (94.97)	10 (5.03)	1	0.0140*
*Bicycle/motorcycle*	2469 (97.74)	57 (2.26)	2.292 (1.152 - 4.560)	
*Personal vehicle*	748 (97.52)	19 (2.48)	2.083 (0.953 - 4.554)	
*Car hire/ school bus*	875 (98.98)	9 (1.02)	5.144 (2.062 - 12.833)	
*Other*	34 (97.14)	1 (2.86)	1.799 (0.223 - 14.512)	
Relationship between caregivers and children				
*Father/mother*	3555 (97.72)	83 (2.28)	1	0.5334
*Relatives/others*	771 (97.35)	21 (2.65)	0.857 (0.528- 1.392)	
Gender of caregiver				
*Male*	916 (98.07)	18 (1.93)	1	0.3409
*Female*	3410 (97.54)	86 (2.46)	0.779 (0.466 - 1.302)	
Nationality of patient				
*Thai*	4296 (97.64)	104 (2.36)	1	0.9801
*Other*	30 (100.00)	0 (0.00)	- ^[2]^	
Ethnicity of patient				
*Thai*	4244 (97.61)	104 (2.39)	1	0.9784
*Other*	82 (100.00)	0 (0.00)	- ^[2]^	
Religion of patient				
*Buddhist*	4057 (97.71)	95 (2.29)	1	0.3140
*Christianity/ Islam/ other*	269 (96.76)	9 (3.24)	0.700 (0.349 - 1.402)	
Age of caregiver (years)				
*≤30*	686 (98.42)	11 (1.58)	1	0.1474
*>30*	3640 (97.51)	93 (2.49)	0.628 (0.334 - 1.179)	
Education level of caregiver				
*None*	194 (97.29)	6 (2.71)	1	0.0002*
*Elementary school*	644 (98.17)	12 (1.83)	1.498 (0.555 - 4.039)	
*Primary school*	814 (95.43)	39 (4.57)	0.582 (0.243 - 1.394)	
*Secondary school*	658 (97.48)	17 (2.52)	1.080 (0.421 - 2.774)	
*High school/vocational certificate*	1160 (98.64)	16 (1.36)	2.023 (0.783 - 5.229)	
*Associate’s Degree /Vocational Certificate/* *bachelor's degree or above*	835 (98.35)	11 (1.65)	1.664 (0.632 - 4.382)	
Caregiver’s occupation				
*Unemployed/ retired/ student*	374 (95.65)	17 (4.35)	1	0.0188*
*Agriculture*	992 (96.97)	31 (3.03)	1.455 (0.796 - 2.660)	
*Manufacturing*	165 (98.80)	2 (1.20)	3.750 (0.857 - 16.418)	
*Worker/laborer*	1789 (98.13)	34 (1.87)	2.392 (1.323 - 4.327)	
*Salesperson*	484 (97.38)	13 (2.62)	1.693 (0.812 - 3.529)	
*Government officer/ state enterprise/ office* *worker/ other*	522 (98.68)	7 (1.32)	3.390 (1.392 - 8.257)	
Marital status of parents				
*Married*	3502 (97.93)	74 (2.07)	1	0.0108*
*Separated*	513 (97.53)	13 (2.47)	0.834 (0.459 - 1.514)	
*Divorced*	178 (95.70)	8 (4.30)	0.470 (0.223 - 0.990)	
*Widowed*	107 (93.86)	7 (6.14)	0.323 (0.145 - 0.718)	
*Unspecified*	26 (92.86)	2 (7.14)	0.275 (0.064 - 1.178)	
Family income/month (baht)				
*≤5,000*	598 (96.92)	19 (3.08)	1	0.0351*
*5,001–10,000*	1674 (97.16)	49 (2.84)	1.085 (0.634 - 1.859)	
*≥10,001*	2054 (98.28)	36 (1.72)	1.813 (1.032 - 3.184)	
Type of family accommodation				
*Own a house*	3638 (97.80)	82 (2.20)	1	0.1512
*Rent*	688 (96.90)	22 (3.10)	0.705 (0.437 - 1.136)	

Most students (97%) continued their education regardless of who they lived with. About 98.1% of students who continued their education lived with male primary caregivers, whereas 97.5% lived with female primary caregivers. A total of 97.6% of Thai patients and 100% of non-Thai patients continued their education. The age, education, and occupation of caregivers were not statistically associated with the decision of the patient to continue their education. Regarding marital status, most parents were married, followed by divorced parents (OR=0.470; 95% CI: 0.223-0.990), while there were significantly fewer widowed parents (OR=0.323; 95% CI: 0.145-0.718).

About 96.9% of households had an average monthly income of <5,000 baht, 97.2% made 5,001-10,000 baht, and 98.3% made >10,000 baht. There were significant differences between households making <5,000 baht and those making >10,000 baht (OR=1.8; 95% CI: 1.032-3.184). Income was not significantly related to education continuation.

Factors associated with education continuation were illness type (more chronic patients discontinued their education), hospitalization period (the longer the stay, the greater the chance of discontinuing education), distance from home to school, transportation type, and factors related to caregivers (i.e., education level, occupation, and marital status).

## Discussion

The 36 learning centers in this study were located in primary or secondary hospitals that had accepted patients from local hospitals in the area. Most patients had chronic illnesses, mostly related to hematology, cancer, and heart disease. This study’s results align with those of previous studies ^10, 11, 13, 16, 17^ of tertiary hospitals. About 45% of patients had common illnesses associated with gastroenterology, respiratory disorders, and accidents. The 282 children in the developmental disorder group had attention deficit hyperactivity disorder, intellectual disability, autism, and learning difficulties.

Compared to research ^10^ conducted in 2000, the number of children with developmental disorders at the learning centers had increased. This is perhaps attributable to patients with physical and developmental disabilities now having better access to education. The average ages of patients with chronic and common illnesses, and developmental disorders were 9.65 years (7.10-12.44), 8.69 years (6.17-11.19), and 8.85 years (6.90-11.11), respectively. Children with chronic illnesses were more likely to discontinue education because of limitations imposed by their health conditions, namely, a high risk of becoming infected or receiving treatment during the academic year. In such cases, modified education plans were introduced. Distance learning, facilitated by technology, is recommended to help patients to keep up with classes, and stay in touch with friends, which would reduce awkwardness when they returned to school. Mobile education can overcome teacher-related limitations, and it has the advantage of allowing children to select lessons for themselves. Other factors that deprived children to attend school in this study were distance from home to school, type of transportation, education level of parents, marital status, and income. However, other factors such as nationality, ethnicity or religion had no relationship with the continuation of education statistically. The results of this study was different from the a previous study ^11^; travel distance and types of transportation in the city did not affect education as much transportation in the country.

Teachers’ roles must be adjusted in the assessment guidelines ^18– 20^ and they must be able to work with patients who have different physical and psychological conditions and education. Teachers should facilitate extra lessons for patients through special classes or e-learning, and help them pass their exams. International research studies ^13, 21, 22^ have found that “bibliotherapy” is an effective method to for reducing anxiety, and developing skills related to learning, emotion regulation, and social interaction.

We found that parents perceived their child’s chronic illnesses as moderately severe to seriously severe. For children with common illnesses, parents found that these illnesses were not moderately severe. Most parents of children with chronic and common illness highly recognized the importance of social modification, they always kept the school updated and were highly confident that the school could provide an appropriate plan for their children. Parents of children with developmental disorders were moderately to highly confident that their child was able to build relationship with friends. Teaching and psychological skills of the teachers were needed because each patient had a different physical condition, education level and psychological condition. They must be able to help the patients reduce anxiety, adjust to their health condition and give appropriate advice. Learning activities could be given anywhere outside the classroom to encourage a learning atmosphere.

In this study, the satisfaction survey of children at the learning center gave a high score of 4.21-5.00 (mean = 4.26, SD = 0.65) for every dimension. The item that was rated the highest score was that the children enjoyed participating in the activities. The item with the second highest score was that there was interesting and up-to-date content (for example, EDLTV is used in teaching according to international standard of the Ministry of Education). The third highest scoring item was that the activities were appropriate for learning (mean = 4.18, SD =0.79). Satisfaction in being part of fun and appropriate activities was rated the fourth highest item, while the item with the lowest score was that there was a variety of material and adequate facilities used in the activities (mean = 4.11, SD = 0.74).

The collaboration between learning centers and schools to monitor and refer patients, establishes a long-term plan and provide appropriate support help the patients a lot. The Cochrane library ^13, 21^ suggests promoting a law that would foster combined work by multidisciplinary teams from hospitals, families, schools, and communities. This way, services could be developed holistically ^22^, and children would be encouraged to continue their education. Social and society family burdens would be reduced, which could achieve the objective of children growing up to be valuable assets of the country.

## Conclusion

Most child patients in the learning centers assessed in this study in Thailand decided to continue their education. Factors that were statistically related to discontinuing education were illnesses type, distance from home to school, transportation type, education level, marital status and family income. Appropriate educational management plans are needed to provide for these children. Parents had positive attitudes about illness and education. Children’s satisfaction with the learning center was high.

### Key messages

- A good education system should allow all children to have equal access to education, including vulnerable children.
- Improvement in technology supports distance learning, which combines patient ‘healthcare, education, and social life’, thus enhancing learning equity, and helping them to return to a normal life after reentering society.
- The factors affecting education continuation should be recognized and considered in education planning for sick children.
- Satisfaction should include every dimension of services for quality improvement.
- The support from Special Education Act and multi-disciplinary team will lead to successful hospital learning centers

### Limitations

The study population was limited to children at 36 hospital learning centers in Thailand. Respondents from various institutes in other contexts should be included to increase diversity. Since different hospitals provide different types and level of services for patients with different needs, future studies should include learning centers in different types of hospitals throughout Thailand such as primary, secondary and tertiary care facilities.

## Data availability

### Underlying data

This project contains the following underlying data:

Figshare: Survey data record of 36 learning centers, Thailand https://doi.org/10.6084/m9.figshare.13140659.v1 ^14^

### Extended data

Figshare: English survey.pdf, https://doi.org/10.6084/m9.figshare.8208338.v2 ^23^

This project contains the following extended data:

- Copy of the survey with English translation.

Data are available under the terms of the Creative Commons Attribution 4.0 International license (CC-BY 4.0).

## References

[1] Annunziato RA Rakotomihamina V Rubacka J : Examining the effects of maternal chronic illness on child well-being in single parent families. *J Dev Behav Pediatr.* 2007;28(5):386–91. 10.1097/DBP.0b013e3181132074 18049321

[2] Atheros T Arpaichiratana C : Model of continuing care foe chronically ill children. *Thai Journal of Nursing Council.* 2011;26(special issue):112–25.

[3] Enderlein G : Daniel, Wayne W.: Biostatistics – A Foundations for Analysis in the Health Sciences. Wiley & Son New York – Chichester – Brisbane – Toronto – Singapore, 6th ed. 1995, 780 S., 58. - , ISBN 0 – 471 – 58852-0 (cloth). *Biom J.* 1995;37(6):744. 10.1002/bimj.4710370610

[4] Law Office of the Basic Education Commission Ministry of Public Health: Education Provision for Persons with Disabilities Act B.E. 2551 and 12 subordinate legislations.Special Education Bureau 2008; (Accessed: 27 January 2020). Reference Source

[5] Pompimon J : A study of the organization programs for young chronically ill in-patient children.Bangkok: Chulalongkorn University. 2000.

[6] Zuckerman B : Developmental and Behavior Pediatrics: a handbook for primary care. 2 ^nd^edn. Philadelphia: Lippincott William & Wilkins,2005;152–7. Reference Source

[7] Wijlaars LPMM Gilbert R Hardelid P : Chronic conditions in children and young people: learning from administrative data. *Arch Dis Child.* 2016;101(10):881–5. 10.1136/archdischild-2016-310716 27246068 PMC5050282

[8] Lum A Wakefield CE Donnan B : Understanding the school experiences of children and adolescents with serious chronic illness: a systematic meta-review. *Child Care Health Dev.* 2017;43(5):645–62. 10.1111/cch.12475 28543609

[9] Council for Exceptional Children Division of Physical, Health an Multiple Disabilities: School Success for children experience chronic illness: national recommendations for addressing global barriers. *Physical Dis.* 2017;36(2):8–15. 10.14434/pders.v36i2.24106

[10] Stoll BJ Hansen NI Bell EF : Neonatal outcomes of extremely preterm infants from the NICHD Neonatal Research Network. *Pediatrics.* 2010;126(3):443–56. 10.1542/peds.2009-2959 20732945 PMC2982806

[11] Yunak R : Interviewing children with chronic illness in qualitative research. *J Nurs Health Sci.* 2012;6:1–11.

[12] LoCasale-Crouch J Johnson B : Transition from pediatric to adult medical care. * Adv chronic kidney Dis.* 2005;12(4):412–7. 10.1053/j.ackd.2005.07.004 16198281

[13] Fuengfoo A Leelathanaporn S Mekrungcharas T : Effectiveness of the Hospital Learning Center (Queen Sirikit National Institute of Child Health): Satisfaction with service and parents’ attitudes towards children’s illness [version 1; peer review: 2 approved, 1 approved with reservations] *F1000Res.* 2019;8:1616. (Accessed: 15 March 2020). 10.12688/f1000research.18846.1 32477495 PMC7218477

[14] Fuengfoo A : Survey data record of 36 learning centers, Thailand. *figshare.* Dataset.2020. 10.6084/m9.figshare.13140659.v1

[15] Daniel WW : Biostatistics:A Foundation of Analysis in the Health sciences.(6th ed.)John Willey & Sons,Inc.1995. Reference Source

[16] Center for Chronic Disease Prevention and Control: What are chronic and noncommunicable diseases?Ottawa, ON: Public Health Agency of Canada. (Accessed: 18 August 2018). Reference Source

[17] Special Education Bureau, Ministry of Education: Guidelines for Performance Evaluation of Learning Center in Hospital for Chronically Ill Patients. Bangkok: Office of the Basic Education Commission 2015; (Accessed: 13 January 2020). Reference Source

[18] Bell MF Bayliss DM Glauert R : Chronic Illness and Developmental Vulnerability at school Entry. *Pediatric.* 2016;137(5):e20152475. 10.1542/peds.2015-2475 27244787

[19] Special Education Bureau, Ministry of Education: ‘Practice Guideline for Teachers in Chronic Children Learning Center in Hospital Bangkok: Office of the Basic Education Commission.2015; (Accessed: 13 January 2020). Reference Source

[20] Van Cleave J Gortmaker SL Perrin JM : Dynamic of obesity and chronic health conditions among children and youth. *JAMA.* 2010;303(7):623–30. 10.1001/jama.2010.104 20159870

[21] Pugbunmee R : Chronically ill adolescents: impact on self-care. *Rama Nurse.* 1997;3:103–9.

[22] World Health Organization: innovative care for chronic conditions: building blocks for action.Geneva, Switzerland: world Health Organization2002; (Accessed: 15 October 2018). Reference Source

[23] Fuengfoo A : English Survey.pdf. *figshare.* Figure.2019. 10.6084/m9.figshare.8208338.v2

