# Supplementary material for: Effectiveness of 36 hospital learning centers in Thailand: continuation of child patient education, parent attitudes toward child’s illness and service satisfaction
Source: F1000Res. 2022 Apr 13;9:1446. Originally published 2020 Dec 11. [Version 2] doi: 10.12688/f1000research.26599.2 (PMC11329862; doi:10.12688/f1000research.26599.2)
Supplement: Peer review history

# Reviewer report: Reviewer response for version 2

**DOI:** 10.5256/f1000research.123004.r295433

**Published:** 2024-09-09

**Authors:** Nutthaporn Chandeying

**Affiliations:** 1 Faculty of Medicine Vajira Hospital, Navamindradhiraj University, Bangkok, Thailand

**Reviewer comment**

**Abstract **

1. A Correct subject-verb agreement throughout the manuscript. For example, "Satisfaction score towards the services at the learning centers were high" should be "Satisfaction scores towards the services at the learning centers were high."
2. Improve clarity and conciseness. For example, "Parents were less worried about children with common illness and more concerned about education of children with chronic diseases and children with disabilities" can be revised to "Parents were less worried about children with common illnesses and more concerned about the education of children with chronic diseases and disabilities."
3. Use correct terminology consistently. For example, "About 99.8% of patients with common illnesses continued their education, followed by 99.3% of disabled children, and 95.9% of chronic patients." Consider revising to "About 99.8% of children with common illnesses continued their education, followed by 99.3% of children with disabilities, and 95.9% of children with chronic illnesses."

**Introduction **

1. There is inconsistency in the use of terms, such as "chronic diseases" and "chronic illnesses." Choose one term and use it consistently throughout the manuscript to avoid confusion.
2. The background lacks detailed information on how the QSNICH and other learning centers operate and the specific roles of different ministries. Provide more details about the operation of these centers, including the roles of the Ministry of Education, Ministry of Public Health, and Ministry of Science and Technology. Explain how the collaboration works and the specific contributions of each ministry.
3. There are several grammatical issues that need attention, such as subject-verb agreement and punctuation. Revise sentences for grammatical correctness.

- "All of the operational process needs to follow Clinical Practice Guideline (CPG) and pass an external audit from the third party" should be "All operational processes need to follow Clinical Practice Guidelines (CPG) and pass an external audit by a third party."

 - Original: "Today medical advancements have increased the survival rate of children with chronic diseases to 90%, reducing mortality rates and complications1, and increasing life expectancy by as much as 20 years longer2."

 Issue: Comma needed after "Today"; "as much as 20 years longer" is redundant.

 Revision: "Today, medical advancements have increased the survival rate of children with chronic diseases to 90%, reducing mortality rates and complications, and increasing life expectancy by up to 20 years."

 - Original: "The UN Convention of the Rights of the Child states that every child has the right to be protected and receive equal education9."

 Issue: "of" should be "on."

 Revision: "The UN Convention on the Rights of the Child states that every child has the right to be protected and receive equal education."

 - Original: "Similar learning centers in other countries involve the coordination of multidisciplinary teams to enhance social and learning activities or sick children."

 Issue: "or" should be "for."

 Revision: "Similar learning centers in other countries involve the coordination of multidisciplinary teams to enhance social and learning activities for sick children."

 - Original: "Hospital Learning Centers for sick children uses standardized curriculum established by Ministry of Education applicable for children at all education levels: pre-kindergarten, primary, secondary, tertiary levels, as well as vocational education."

 Issue: "uses" should be "use"; "established by Ministry of Education" needs an article.

 Revision: "Hospital Learning Centers for sick children use a standardized curriculum established by the Ministry of Education, applicable for children at all education levels: pre-kindergarten, primary, secondary, tertiary, and vocational education."

 - Original: "This curriculum is a collaboration between MOU partners: Ministry of Education, Ministry of Public Health and Ministry of Science and Technology."

 Issue: Comma needed before "and."

 Revision: "This curriculum is a collaboration between MOU partners: the Ministry of Education, the Ministry of Public Health, and the Ministry of Science and Technology."

 4. The objectives section can be made clearer and more precise. Clearly state the specific objectives of the study in bullet points or a numbered list for better readability. For example: This study extends previous research on the QSNICH learning center's effectiveness, focusing on factors affecting further education for children with special needs. The aims are To determine the effectiveness of 36 network learning centers across Thailand in providing continued education for pediatric patients.

1. To identify factors associated with the continuation of education for pediatric patients.
2. To evaluate parents' attitudes toward their children's illnesses and education.
3. To assess service satisfaction at the learning centers.

**Methods **

1. Lack of clarity in the description of statistical methods. Clearly describe the statistical methods used, including the rationale for choosing chi-square tests and t-tests. Explain how data were managed.
2. Several grammatical errors, including subject-verb agreement and punctuation issues. Revise sentences for grammatical correctness. Ensure subject-verb agreement, proper punctuation, and correct use of articles as the followings:

- Original: "This research was conducted in a form of natural experimental study which variables are influenced by nature or factors outside of the researchers' control."

 Issue: "in a form of" should be "as a"; "which variables are" should be "where variables are."

 Revision: "This research was conducted as a natural experimental study where variables are influenced by nature or factors outside of the researchers' control."

 - Original: "Cross-sectional design has been implemented in this study where a group of population from all the 36 learning centers in the hospitals across Thailand was selected based on the inclusion criteria set for the study: types of illnesses, patient's and parent's demographic information in order to measure the effectiveness of the project and to examine the factors correlating with continuing education of children under HSH project, patient's attitudes and patient's satisfaction towards HSH project."

 Issue: "Cross-sectional design has been implemented" should be "A cross-sectional design was implemented"; "group of population" should be "group of participants"; "in the hospitals" is redundant.

 Revision: "A cross-sectional design was implemented in this study, selecting a group of participants from all 36 learning centers across Thailand based on the inclusion criteria set for the study: types of illnesses, patients' and parents' demographic information. The study aimed to measure the effectiveness of the project and examine factors correlating with the continuation of education for children under the HSH project, as well as patients' attitudes and satisfaction with the HSH project."

 - Original: "The total number of pediatric patients from 36 learning centers at the hospitals was 30,000 (n=30,000), and the incidence of children who went back to school was 80% (p=0.8))."

 Issue: Redundant parentheses; awkward phrasing.

 Revision: "The total number of pediatric patients from the 36 learning centers was 30,000 (n=30,000), with an incidence of 80% (p=0.8) for children returning to school."

 - Original: "The selection of a sample in this study was a voluntary response sample from all 36 learning centers in Hospitals across Thailand."

 Issue: "Hospitals" should not be capitalized; awkward phrasing.

 Revision: "The sample selection in this study was a voluntary response sample from all 36 learning centers across hospitals in Thailand."

 - Original: "That is, after the instruction was given to the participants, they were able to participate in the study voluntarily."

 Issue: Redundant phrasing; "instruction" should be "instructions."

 Revision: "After receiving instructions, participants were able to voluntarily participate in the study."

 - Original: "If the number of participants were more than expected population, a random sample selection would be implemented."

 Revision: "If the number of participants exceeded expectations, a random sample selection would be implemented."

 - Original: "The session was audio recorded for data analysis."

 Issue: "audio recorded" should be "audio-recorded."

 Revision: "The session was audio-recorded for data analysis."

 - Original: "In order to access the data, a permission from the Human Ethics Committee of the Ministry of Public Health that oversees all 36 network hospitals must be approved."

 Issue: "a permission" should be "permission"; "must be approved" should be "was required."

 Revision: "To access the data, permission from the Human Ethics Committee of the Ministry of Public Health overseeing all 36 network hospitals was required."

 - Original: "Pediatric patients age between 4-18 years who are attending learning centers at the hospital;"

 Issue: "age" should be "aged."

 Revision: "Pediatric patients aged between 4-18 years who are attending learning centers at the hospital;"

 - Original: "Pediatric patients who have attended learning centers more than 2 times;"

 Issue: "more than 2 times" should be "more than twice."

 Revision: "Pediatric patients who have attended learning centers more than twice;"

 - Original: "pediatric patients with chronic illnesses who are admitted at the hospital or needed consecutive follow-up."

 Issue: "admitted at the hospital" should be "admitted to the hospital"; "needed" should be "need."

 Revision: "Pediatric patients with chronic illnesses who are admitted to the hospital or need consecutive follow-up."

 - Original: "pediatric patients with serious illness and life-threatening conditions."

 Issue: "serious illness" should be "serious illnesses."

 Revision: "Pediatric patients with serious illnesses and life-threatening conditions."

 - Original: "The survey was divided into four parts: (1) demographic information of the children and parents (gender, age, cause of admission and length of stay (Table 1)), (2) information about the patient-parent relationship, and (3) satisfaction with service at the learning centers (measured using Likert scales of 1=unsatisfied, 2=poor, 3=fair, 4=satisfied, 5=very satisfied)."

 Issue: "four parts" but only lists three; misplaced parentheses.

 Revision: "The survey was divided into four parts: (1) demographic information of the children and parents (gender, age, cause of admission, and length of stay; see Table 1), (2) information about the patient-parent relationship, (3) satisfaction with service at the learning centers (measured using Likert scales of 1=unsatisfied, 2=poor, 3=fair, 4=satisfied, 5=very satisfied), and (4) attitudes toward the HSH project."

 - Original: "Chi-square tests and t-tests were used to assess the variables associated with education continuance at the learning center."

 Issue: "education continuance" should be "continuation of education."

 Revision: "Chi-square tests and t-tests were used to assess the variables associated with the continuation of education at the learning centers."

**Results **

1. The presentation of statistical data is inconsistent and sometimes unclear. Percentages are used in some places without clear context or comparison. Present statistical data in a more structured format, such as tables or bullet points, to clearly show the comparison between different groups. Ensure that all percentages are accompanied by the relevant sample size or context as the followings:

- Original: "There were a total of 4,430 caregivers: 82.94% were caregivers for patients with common illnesses, 82.68% chronic illnesses, and 72.34% developmental disorders."

 Suggestion: "There were a total of 4,430 caregivers: 3,673 (82.94%) for patients with common illnesses, 3,660 (82.68%) for patients with chronic illnesses, and 3,206 (72.34%) for patients with developmental disorders."

 - Inconsistent and unclear presentation of caregiver demographics. Original: "Most caregivers were women (75.74%, 80.83%, and 82.62%, respectively), of Thai nationality (99.26%, 99.33%, and 99.65%, respectively), and Thai ethnicity (97%, 98.83%, and 99.65%, respectively)."

 Suggestion: "Most caregivers were women: 3,356 (75.74%) for patients with common illnesses, 3,636 (80.83%) for patients with chronic illnesses, and 3,656 (82.62%) for patients with developmental disorders. The majority were of Thai nationality: 4,396 (99.26%), 4,410 (99.33%), and 4,414 (99.65%), respectively, and of Thai ethnicity: 4,293 (97%), 4,379 (98.83%), and 4,414 (99.65%), respectively."

 - Original: "About 57.0% traveled to school by bicycle/motorcycle, 17.3% by private car, and 20.0% by public transportation; the means of transportation remained almost the same before and after being treatment at the center."

 Suggestion: "Approximately 2,525 (57.0%) traveled to school by bicycle/motorcycle, 766 (17.3%) by private car, and 886 (20.0%) by public transportation; the means of transportation remained almost the same before and after treatment at the center."

1. Several sentences contain grammatical errors, awkward phrasing, and inconsistencies in verb tenses. Revise sentences for grammatical correctness. Ensure subject-verb agreement, proper punctuation, and consistent use of verb tenses. For example:

Original: "Most caregivers were women (75.74%, 80.83%, and 82.62%, respectively), of Thai nationality (99.26%, 99.33%, and 99.65%, respectively), and Thai ethnicity (97%, 98.83%, and 99.65%, respectively)."

 Revision: "Most caregivers were women (75.74%, 80.83%, and 82.62%, respectively), of Thai nationality (99.26%, 99.33%, and 99.65%), and of Thai ethnicity (97%, 98.83%, and 99.65%)."

**Discussion **

1. Several sentences contain grammatical errors, awkward phrasing, and inconsistencies in verb tenses. Revise sentences for grammatical correctness. Ensure subject-verb agreement, proper punctuation, and consistent use of verb tenses. For example:

Original: "Mobile education can overcome teacher-related limitations, and it has the advantage of allowing children to select lessons for themselves."

 Revision: "Mobile education can overcome teacher-related limitations and has the advantage of allowing children to select lessons for themselves."

1. Inconsistent presentation of statistical data and lack of context for percentages.

Present statistical data in a more structured format, such as tables or bullet points, to clearly show comparisons between different groups. Ensure that all percentages are accompanied by the relevant sample size or context. For example:

 Original: "In this study, the satisfaction survey of children at the learning center gave a high score of 4.21-5.00 (mean = 4.26, SD = 0.65) for every dimension."

 Revision: "In this study, the satisfaction survey of children at the learning center showed high scores in all dimensions, with an overall mean score of 4.26 (SD = 0.65). The highest-rated item was enjoyment of activities (mean = 4.31), followed by interest in content (mean = 4.27)."

1. Limited discussion of how the study's findings compare with existing literature, and lack of integration of previous studies' findings. More thoroughly compare and contrast the study's findings with those of previous studies. Discuss how this study supports, contradicts, or expands upon existing knowledge. For example:

Original: "This study's results align with those of previous studies10,11,13,16,17 of tertiary hospitals."

 Revision: "This study's results align with those of previous studies conducted in tertiary hospitals10,11,13,16,17, which also found high rates of chronic illnesses related to hematology, cancer, and heart disease. However, unlike previous studies, our findings highlight an increase in developmental disorder diagnoses, suggesting improved access to education for these patients."

Is the work clearly and accurately presented and does it cite the current literature?

Yes

If applicable, is the statistical analysis and its interpretation appropriate?

Yes

Are all the source data underlying the results available to ensure full reproducibility?

Partly

Is the study design appropriate and is the work technically sound?

Yes

Are the conclusions drawn adequately supported by the results?

Yes

Are sufficient details of methods and analysis provided to allow replication by others?

Yes

Reviewer Expertise:

Implementation science of maternal and child health

I confirm that I have read this submission and believe that I have an appropriate level of expertise to confirm that it is of an acceptable scientific standard.

# Reviewer report: Reviewer response for version 2

**DOI:** 10.5256/f1000research.123004.r304892

**Published:** 2024-08-16

**Authors:** Banchaun Benjasuwantep

**Affiliations:** 1 Department of Pediatrics, Faculty of Medicine, Srinakharinwirot University, Nakhon Nayok, Thailand

The article is about hospital learning center in Thailand. The strength of the article is the number of hospital learning centers and participants. However, there are some suggestions.

**Objectives**

 The effectiveness of hospital learning center which the researcher would like to evaluate is not clear. What are the primary and secondary outcomes?

**Methods**

1. What were criteria to include the hospital learning center to the research? The author should give information of the learning center.
2. As the participant in the study was voluntary, how many children were refuse to be in the research? Did the participants represent actual population?  It might be research bias if there were a lot of children refuse to participate.

**Results**

1. Under the heading of parent-child relationship, the table of the content should be provided.  
2. About table 3, the author mentions that patients who continued their education thought that the lessons in the classroom were interesting, they scored this item higher than the other group did. However, table 3 shows that scoring of “I am learning interesting lessons at the center” for the group of continued education was 4.31 and the group of did not continued education was 4.48. Is this corrected??
3. Table 4 is about the variables to continuation of education which is interesting part of the article. The author should mention more about the variables that were significant different between the children who continue the education and who did not rather than select a few variables. It is a good idea to separate participants into 3 groups and evaluate the variables associate with continuation of education. However, the author can provide more information about the variables which were different between children who continued to educate and who did not and then separate them into 3 groups as previously done.

**Discussion**

1. The last paragraph of page 9, “In this study, the satisfaction survey” should be mentioned in the result section rather than discussion section.
2. Should discuss more about the effectiveness for the learning center.

Is the work clearly and accurately presented and does it cite the current literature?

Partly

If applicable, is the statistical analysis and its interpretation appropriate?

Partly

Are all the source data underlying the results available to ensure full reproducibility?

Partly

Is the study design appropriate and is the work technically sound?

Partly

Are the conclusions drawn adequately supported by the results?

Partly

Are sufficient details of methods and analysis provided to allow replication by others?

Partly

Reviewer Expertise:

Developmental Behavioral pediatrics

I confirm that I have read this submission and believe that I have an appropriate level of expertise to confirm that it is of an acceptable scientific standard.

# Reviewer report: Reviewer response for version 1

**DOI:** 10.5256/f1000research.29363.r118876

**Published:** 2022-01-17

**Authors:** Shervin Assari, Babak Najand

**Affiliations:** 1 Department of Family Medicine, Charles R Drew University of Medicine and Science, Los Angeles, CA, USA; 2 Charles R. Drew University of Medicine and Science, Los Angeles, CA, USA

This is a single-group interventional study. Here are areas for improvement:

1. The abstract and also the paper should make the design much more clear. Is this a longitudinal one-arm intervention (designed by authors) or is this a natural experiment designed by a system and the authors just conducted the evaluation?
2. The result is not just about attitudes. Many other factors such as socioeconomic status predict child educational success, but the title only mentions attitude.
3. One major problem is that all samples are analyzed together. However, some of these children have physical health problems and some have disabling developmental challenges. These two groups are qualitatively different and cannot be combined. Just controlling for this type as a nominal variable (0/1) does not solve the problem. I think at least for sensitivity analysis, all results should be replicated in a stratified fashion, separately for each of the groups mentioned. 
4. SES and social determinants of health are root causes of health and well-being. The paper, however, does not mention these concepts despite that it measures them.
5. Overall, I think the data are much stronger than the discussion section. Many more areas could be discussed which are left untouched. 
6. These children are educationally heterogeneous. This includes children at kindergarten to much higher levels. Progress in educational programs is highly dependable on the workload required. So, simply controlling for the educational level does not solve this problem. Overall, the high heterogeneity of the sample is a major problem here.
7. Sex/gender is beyond a control variable. Parents may differently invest in their boys and girls (gender norms). Similarly, a child may get different attention and investment depending on the number of siblings. Thus, more attention is needed on the composition of the household beyond marital status.
8. Another important area in this paper is poor eligibility. These poor selection criteria have made it very difficult to know who was in the study. I think the authors need to replicate their findings in groups of patients with more homogenous conditions. 
9. More information is needed on the variation of the adherence to the curriculum across learning centers. How do various centers do their job? Was there any effect on centers? 
10. Participants were nested to centers so they are not independent samples. Please discuss your solution to address this data challenge.

Overall, despite the paper having great potential and focusing on a novel topic that is rarely done by others, authors could do a much better job with inclusion, statistics, and discussion.

Is the work clearly and accurately presented and does it cite the current literature?

Partly

If applicable, is the statistical analysis and its interpretation appropriate?

No

Are all the source data underlying the results available to ensure full reproducibility?

Partly

Is the study design appropriate and is the work technically sound?

Partly

Are the conclusions drawn adequately supported by the results?

Partly

Are sufficient details of methods and analysis provided to allow replication by others?

No

Reviewer Expertise:

I am an expert of child development, social inequalities, and longitudinal data analysis. I also work related to chronic disease and mental health problems.

We confirm that we have read this submission and believe that we have an appropriate level of expertise to state that we do not consider it to be of an acceptable scientific standard, for reasons outlined above.

# Response

**Published:** 2022-03-29

**Authors:** adidsuda fuengfoo

**Affiliations:** Queen sirikit national institute of child health, Thailand

**1. This is a single-group interventional study. Here are areas for improvement: The abstract and also the paper should make the design much more clear. Is this a longitudinal one-arm intervention (designed by authors) or is this a natural experiment designed by a system and the authors just conducted the evaluation?**

 This research was conducted in a form of *natural experimental* study in which variables are influenced by nature or factors outside of the researchers’ control. A cross-sectional design has been implemented in this study where a group of the population is selected based on the inclusion criteria set for the study: types of illnesses, patient’s and parent’s demographic information in order to measure the effectiveness of the project and to examine the factors correlating with continuing education of children under HSH project, patient’s attitudes and patient’s satisfaction towards HSH project. However, a limitation of a cross-sectional study is that it is difficult to identify causal effects and only describe associations between variables.

**2. The result is not just about attitudes. Many other factors such as socioeconomic status predict child educational success, but the title only mentions attitude.**

 Assessment of the Learning Center in Hospital Program was assessed on the function and effectiveness of children with health problems participating in the Learning Center in Hospital Program. Another angle, besides taking into account accuracy, should also consider preferences along the way. Therefore, it is assessed in various dimensions, such as assessing the perspectives of service users in terms of parental attitudes and the satisfaction of sick children participating in the Hospital Learning Center project, which is a secondary objective of this research and is discussed in the information found for the purpose.

**3. One major problem is that all samples are analyzed together. However, some of these children have physical health problems and some have disabling developmental challenges. These two groups are qualitatively different and cannot be combined. Just controlling for this type as a nominal variable (0/1) does not solve the problem. I think at least for sensitivity analysis, all results should be replicated in a stratified fashion, separately for each of the groups mentioned.**

 The research team would like to accept and appreciate the advice that is very helpful. In the issue of data analysis should be separated into groups, diseases because each group of diseases has a different nature and affects learning. The researcher, therefore, brought this issue to further improve in the analysis of the data to separate the groups into three groups: common disease children, chronic disease children, and children with developmental problems, as shown in the table.

**4. SES and social determinants of health are root causes of health and well-being. The paper, however, does not mention these concepts despite that it measures them.**

 Socioeconomic status is a very important part of healthcare and is the foundation for proper health management and lifestyle. It is a valuable recommendation when further analyzed separately. The disease group found that social socioeconomic status affecting admission to continuing education after discharge from the hospital was associated with all disease groups. When analyzed, it was found that there was still a relationship between chronic disease and chronic disease groups with developmental problems because it is a long continuous treatment, and with costs that depend on severity, complexity, and advances in technology, children with chronic and developmental diseases have a better chance of living longer.

**5. Overall, I think the data are much stronger than the discussion section. Many more areas could be discussed which are left untouched. ** 

 The researcher agreed and used the data for further analysis, separating the disease groups. We have added more discussion on factors that remained related to children's continued return to school when analyzing disease groups: parents' education, socioeconomic status, family income, number of siblings, etc.

**6. These children are educationally heterogeneous. This includes children at kindergarten to much higher levels. Progress in educational programs is highly dependable on the workload required. So, simply controlling for the educational level does not solve this problem. Overall, the high heterogeneity of the sample is a major problem here.**

 We agree on the issue of diversity in the classroom as one of the limitations of education in nature experimental study. In a future study, we will include prospective follow-up studies and segmented samples for cross-sectional study to reduce such limitations.

**7. S** **ex/gender is beyond a control variable. Parents may differently invest in their boys and girls (gender norms). Similarly, a child may get different attention and investment depending on the number of siblings. Thus, more attention is needed on the composition of the household beyond marital status.**

 Since gender is an uncontrolled variation, the researcher has selected the factors that are more likely to affect the continuation of children which are the number of siblings and family income. After analyzing separately, the study found that parents with more than 2 children are likely to discontinue education of the child with chronic diseases or with developmental problems. At the same time, families with a monthly income lower than 5,000 baht considered stopping further education of the sick child. With economic constraints, parents may need to think about the cost-effectiveness of sending their child to school. It must be decided that delivering a child without chronic illnesses or developmental delay may be more cost-effective than children with chronic illnesses or developmental delay since education cost is not the only expense under parents’ responsibility, but also healthcare costs or medication costs for the sick child. In contrast, the study found that the number of siblings and hospital length of stay do not correlate with children without chronic illnesses or developmental delay to further education because the normal school system allows them to go back to school after they recover.

**8.** **Another important area in this paper is poor eligibility. These poor selection criteria have made it very difficult to know who was in the study. I think the authors need to replicate their findings in groups of patients with more homogenous conditions.**

 It is a valuable suggestion that gives the researcher a clearer perspective. Therefore, the researcher has divided the groups to select the pediatric patients by separate disease groups and analyze the factors affecting admission to further education in order to create more homogeneous conditions as shown in the table.

**9. More information is needed on the variation of the adherence to the curriculum across learning centers. How do various centers do their job? Was there any effect on centers?**

 Hospital Learning Centers for sick children use a standardized curriculum established by the Ministry of Education applicable for children at all education levels: pre-kindergarten, primary, secondary, tertiary levels, as well as vocational education. This curriculum is a collaboration between MOU partners: the Ministry of Education, Ministry of Public Health, and Ministry of Science and Technology.

 All of the operational processes need to follow the Clinical Practice Guideline (CPG) and pass an external audit from a third party to ensure that the education that is delivered to sick patients in every hospital learning center meets the standard.

**10.** **Participants were nested to centers so they are not independent samples. Please discuss your solution to address this data challenge.**

 The selection of a sample in this study was a voluntary response sample from all 36 learning centers in Hospitals across Thailand. That is, after the instruction was given to the participants, they were able to participate in the study voluntarily. If the number of participants was more than the expected population, a random sample selection would be implemented. In order to access the data, permission from the Human Ethics Committee of the Ministry of Public Health that oversees all 36 network hospitals must be approved.

# Reviewer report: Reviewer response for version 1

**DOI:** 10.5256/f1000research.29363.r78813

**Published:** 2021-02-17

**Authors:** Issarapa Chunsuwan

**Affiliations:** 1 Department of Pediatrics, Faculty of Medicine, Developmental and Behavioral Pediatrics, Thammasat University (TU), Pathum Thani, Thailand

This is continuing research to find ways to promote education for children in hospitals. The hospital school model was further implemented in 36 centers in Thailand in which many children have received services. In this paper, the authors aim to evaluate the effectiveness of the learning centers.

 I have some suggestion as following:

**Introduction part**

- For a clearer understanding, the Authors should give more information about the 36 learning centers, such as how they are settled in hospitals (place, operational time), who are teachers in the centers, how they select pediatric patients to attend the centers (by doctor referral or who else) and how they provide educational service (curriculum, activity)? And also, more information about eDLTV may help readers who are not in Thailand understand it. The Authors may add information about how the QSNICH learning center supervises the other 36 learning centers. 

**Methodology**

- A flow chart that shows how 4430 participants in this study were recruited from 30,000 pediatric patients of the 36 centers could be helpful to show a clear overview of the recruitment process. 
- There are 4430 data in the research rather than 8860. The author can explain that 4430 children included in the research and 4430 parents were interviewed.  
- Definition for classifying common illness from chronic illness should be mentioned (depend on the type of disease or hospital day)
- The questionnaires used in the study should be defined in detail for better understanding, such as numbers of items in the satisfactory scales and how is the total score obtained, what are questions asked in the demographic data and validation process of the questionnaire.

**Results**

- The main research findings on effectiveness may not yet clear to readers. The authors may adjust or add a table that defines the effectiveness of the program. For example, in Table 3, statistical analysis of differences in satisfaction between 2 groups could add more information about effectiveness.  
- In table 4, Is not clear that the heading "continue education" means continue education at a learning center or at a formal school. and also the distance from school means the formal school or learning center. 

**Key messages**

- The main result of this study about effectiveness should be added as the key message.

Is the work clearly and accurately presented and does it cite the current literature?

Partly

If applicable, is the statistical analysis and its interpretation appropriate?

Partly

Are all the source data underlying the results available to ensure full reproducibility?

Partly

Is the study design appropriate and is the work technically sound?

Partly

Are the conclusions drawn adequately supported by the results?

Partly

Are sufficient details of methods and analysis provided to allow replication by others?

Partly

Reviewer Expertise:

Developmental and behavioral pediatrician

I confirm that I have read this submission and believe that I have an appropriate level of expertise to confirm that it is of an acceptable scientific standard.
